# Genome-wide analysis of UDP-glycosyltransferase gene family and identification of members involved in flavonoid glucosylation in Chinese bayberry (*Morella rubra*)

**DOI:** 10.3389/fpls.2022.998985

**Published:** 2022-09-26

**Authors:** Chuanhong Ren, Yunlin Cao, Mengyun Xing, Yan Guo, Jiajia Li, Lei Xue, Chongde Sun, Changjie Xu, Kunsong Chen, Xian Li

**Affiliations:** ^1^Zhejiang Provincial Key Laboratory of Horticultural Plant Integrative Biology, Zhejiang University, Hangzhou, China; ^2^The State Agriculture Ministry Laboratory of Horticultural Plant Growth, Development and Quality Improvement, Zhejiang University, Hangzhou, China

**Keywords:** *Morella rubra*, UGT, anthocyanin, flavonol, UDP-glucosyltransferase

## Abstract

Glycosylation was catalyzed by UDP-glycosyltransferase (UGT) and was important for enriching diversity of flavonoids. Chinese bayberry (*Morella rubra*) has significant nutritional and medical values because of diverse natural flavonoid glycosides. However, information of *UGT* gene family was quite limited in *M. rubra*. In the present study, a total of 152 *MrUGT* genes clustered into 13 groups were identified in *M. rubra* genome. Among them, 139 *MrUGT* genes were marked on eight chromosomes and 13 members located on unmapped scaffolds. Gene duplication analysis indicated that expansion of *MrUGT* gene family was mainly forced by tandem and proximal duplication events. Gene expression patterns in different tissues and under UV-B treatment were analyzed by transcriptome. Cyanidin 3-*O*-glucoside (C3Glc) and quercetin 3-*O*-glucoside (Q3Glc) were two main flavonoid glucosides accumulated in *M. rubra*. UV-B treatment significantly induced C3Glc and Q3Glc accumulation in fruit. Based on comprehensively analysis of transcriptomic data and phylogenetic homology together with flavonoid accumulation patterns, MrUFGT (MrUGT78A26) and MrUGT72B67 were identified as UDP-glucosyltransferases. MrUFGT was mainly involved in C3Glc and Q3Glc accumulation in fruit, while MrUGT72B67 was mainly involved in Q3Glc accumulation in leaves and flowers. Gln375 and Gln391 were identified as important amino acids for glucosyl transfer activity of MrUFGT and MrUGT72B67 by site-directed mutagenesis, respectively. Transient expression in *Nicotiana benthamiana* tested the function of MrUFGT and MrUGT72B67 as glucosyltransferases. The present study provided valuable source for identification of functional UGTs involved in secondary metabolites biosynthesis in *M. rubra*.

## Introduction

Diverse plant secondary metabolites such as flavonoids play important roles in plant development and human health (Yin et al., 2014; Bondonno et al., 2019; Alseekh et al., 2020). Glycosylation usually occurs during later stages in many secondary metabolite biosynthesis pathways. Glycosylation could improve solubility, stability, transferability, and diversity of many plant secondary metabolites like flavonoids (Bowles et al., 2006; Yang et al., 2018; Naeem et al., 2021).

UDP-glycosyltransferase (UGT) family was the largest family in plants among GT super families reported in CAZy^1^ database. It catalyzed glycosylation formation of many small molecules, including flavonoids, hormones, and xenobiotics (Vogt and Jones, 2000; Bowles et al., 2006). With the rapid development in bioinformatics and plant genomics, *UGT* gene families have been identified in many plants, from algae *Chlamydomonas reinhardtii* to vascular plants like *Selaginella moellendorffii* and *Prunus persica* (Caputi et al., 2012; Wu et al., 2017). In model plant *Arabidopsis thaliana*, 107 *UGT* members were identified in genome, and were clustered into 14 groups (A-N) based on phylogenetic relationship analysis (Ross et al., 2001). Subsequently, four new phylogenetic groups, named O, P, Q, and R, that were not presented in *Arabidopsis* were discovered in *Malus* × *domestica* (Caputi et al., 2012), *Zea mays* (Li et al., 2014), and *Camellia sinensis* (Cui et al., 2016). Gene family identification facilitates discovery of functional *UGT* genes. CsUGT78A14 and CsUGT78A15 were found to be involved in astringent taste compounds biosynthesis by analysis of *C*. *sinensis UGT* gene family (Cui et al., 2016). And several UGTs involved in biosynthesis of anti-diabetic plant metabolite Montbretin A were discovered based on *UGT* gene family analysis (Irmisch et al., 2018; Irmisch et al., 2020).

UDP-glycosyltransferase family contains a conserved motif close to C-terminal, named the plant secondary product glycosyltransferase (PSPG) box. Amino acids in PSPG-box were important for glycosyl transfer activity of UGTs (Shao et al., 2005; Offen et al., 2006; Osmani et al., 2009). For example, last amino acid residue of PSPG-box for UDP-glucosyltransferases usually was glutamine (Gln), and examples include VvGT1 (Ford et al., 1998), MdUGT71B1 (Xie et al., 2020), and PpUGT78T3 (Xie et al., 2022). However, other amino acids could also influence UGT sugar donor preference and more UGTs with different functions should be identified to elucidate the mechanism of sugar donor preference of UGTs.

Chinese bayberry (*Morella rubra*), a member of the Myricaceae, has significant nutritional and medical values due to high content of diverse natural flavonoids such as flavonol glycosides and anthocyanins (Sun et al., 2013; Zhang et al., 2015; Liu et al., 2022). It was reported that flavonoid-rich extracts of fruit and leaves had diverse bioactivities such as antioxidant (Sun et al., 2013; Yan et al., 2016), anti-diabetes (Sun et al., 2013; Liu et al., 2020), and anti-cancer (Sun et al., 2012). However, information of *UGT* gene family and identification of UGTs related to flavonoid glycosylation in *M. rubra* were limited. Recently, both transcriptome and genome information with high-quality have been published in *M. rubra* (Feng et al., 2013; Jia et al., 2019), which makes identification of *UGT* gene family in this plant available.

In the present study, a comprehensive genome-wide identification of *UGT* gene family was carried out in *M*. *rubra*. A total of 152 MrUGT putative proteins were identified from *M. rubra* genome. Genome-wide analysis was performed including phylogenetic relationship, gene structure, chromosome distribution, and gene duplication. Furthermore, expression patterns of *MrUGT* genes were analyzed by Ribonucleic Acid (RNA)-seq in different tissues and ultraviolet (UV) B-treated fruit. Base on *MrUGT* gene family analysis, MrUFGT (MrUGT78A26) and MrUGT72B67 were identified as flavonoid 3-*O*-glucosyltransferases by *in vitro* and *in vivo* investigations. In addition, important amino acids were identified for glucosyl transfer activity of MrUFGT and MrUGT72B67 by site-direct mutagenesis.

## Materials and methods

### Identification and phylogenetic analysis of *MrUGT* gene family

A Hidden Markov Model (HMM) profile for UGT (PF00201) downloaded from Pfam^2^ database was used as a query file to identify UGT proteins in *M. rubra* genome using simple HMM search program in TBtools (Jia et al., 2019; Chen et al., 2020). Multiple EM for Motif Elicitationv (MEME, suite 5.0.3) website and CDD^3^ were used to check completeness of MrUGT sequences. Incomplete coding sequences were manually corrected based on RNA-Seq database (PRJNA714192). MrUGT protein sequences and other plant UGTs were aligned with MUSCLE program. Phylogenetic tree was constructed using neighbor-joining method in MEGA-X with 1000 bootstrap replicates. Genbank accession numbers could be found in Supplementary Table 1. Multiple sequence alignment was carried out using MUSCLE program between MrUGTs and other glucosyltransferases. Sequence alignment was visualized using GeneDoc software.

### Analysis of conserved motif and gene structure

Conserved motifs in MrUGT proteins were analyzed by simple MEME Wrapper program in TBtools with default parameters. Results of conserved motifs were visualized by TBtools (Chen et al., 2020). Sequences of conserved motifs were visualized by WebLogo^4^ website (Crooks et al., 2004). Intron-exon map of *MrUGT* was constructed according to genome annotation file. Gene Structure Display Server 2.0^5^ was used to investigate intron-exon structure in *MrUGT* gene family using sequence format (Hu et al., 2014).

### Chromosome distribution and syntenic analysis of *MrUGT* gene family

Gene Location Visualize program of TBtools was used to investigate and visualize chromosome distribution of *MrUGT* genes according to genome annotation file (Chen et al., 2020). To investigate the evolutionary relationship between MrUGTs and UGTs of other species, synteny analysis was performed within three Rosids species, i.e., *Arabidopsis*, walnut (*Juglans regia*), and peach (*P. persica*). Synteny relationship was analyzed by One Step MCScanX program and visualized by DualSyntePlot program with the help of TBtools (Chen et al., 2020). DupGen_finder program was used to analyze gene duplication events in *M. rubra* genome (Qiao et al., 2019).

### Chemicals reagents

Quercetin (Q), kaempferol (K), quercetin 3-*O*-glucoside (Q3Glc), flavanones (naringenin and hesperetin), flavanols (epicatechin and catechin), flavones (apigenin and luteolin), and isoflavones (genistein and daidzein) were purchased from Aladdin (Shanghai, China). Cyanidin (C) and pelargonidin (P) were purchased from Extrasynthese (Lyon, France). Gradient grade for liquid chromatography of methanol and acetonitrile as well as cyanidin 3-*O*-glucoside (C3Glc) were purchased from Sigma-Aldrich (St. Louis, MO, USA). UDP-glucose (UDP-Glc), UDP-rhamnose (UDP-Rha), and UDP-galactose (UDP-Gal) were obtained from Yuanye Bio-Technology Co., Ltd., (Shanghai, China).

### Plant materials and ultraviolet-B treatment

Flowers, leaves, and fruit of different development stages of *M. rubra* cv. Biqi were obtained from an orchard in Lanxi (Zhejiang, China). Four fruit development stages were: S1 for 45 days after flowering (DAF); S2 for 75 DAF; S3 for 80 DAF; S4 for 85 DAF. All materials were uniform in size and free from mechanical damage. Samples were cut into small pieces, frozen with liquid nitrogen immediately, and stored at −80°C for further analysis. All samples were collected for three biological replicates.

UV-B treatment was carried out as reported (Xie et al., 2020) with some modifications. Treatments were carried out at different layers in the same climatic chambers under controlled conditions with a relative humidity of 90–96% and constant temperature at 20°C. Fruit of ‘Biqi’ cultivar at 70 DAF were selected to treated with UV-B irradiation. Fruit were divided into two groups, and one group was exposed to UV-B irradiation (280–315 nm, 50 μW cm^–2^) for 2 and 6 days. Fruit of control group were put in the dark. Incubator was covered with black cloth to avoid light pollution. Three biological replicates were used and each replicate contained five to eight fruits.

### RNA-seq and gene expression

Total RNA was isolated using cetyltrimethylammonium bromide (CTAB) method as reported (Feng et al., 2013). Integrity of total RNA was detected using nanodrop and gel electrophoresis. RNA-Seq of UV-B-treated fruit was carried out by Novogene Technology Co., Ltd. (Beijing, China). RNA-Seq platform was Illumina Novaseq. Library was prepared using NEBNext Ultra RNA Library Prep Kit for Illumina. Gene expression levels were assessed by FPKM values. Different expression analysis was carried out using DESeq2 (1.20.0). Heatmap of transcript profiles was presented by TBtools (Chen et al., 2020). Gene expression was performed by reverse transcription quantitative PCR (RT-qPCR) as reported (Cao et al., 2019) using primers showed in Supplementary Table 2. *Actin* gene (*MrACT*, GQ340770) was used as internal reference gene. Relative gene expression was calculated using 2^–ΔΔ*Ct*^ method.

### HPLC analysis of flavonoid glycosides

Flavonoid glycosides were extracted and analyzed as reported (Downey et al., 2007; Cao et al., 2019) with some modifications. Sample powder with 0.1 g was sonicated in 1 ml 50% methanol/water (v/v) for 30 min at room temperature. After centrifugation at 12,000 rpm for 15 min, precipitates were extracted one more time. Both supernatants were combined and then analyzed by high-performance liquid chromatography (HPLC) after centrifugation at 12,000 rpm for 15 min as previous reported (Cao et al., 2019). Standard curves were used to quantitate Q3Glc at 350 nm and C3Glc at 520 nm.

### Protein recombination and purification

Coding sequences of *MrUFGT* and *MrUGT72B67* were subcloned into expression vector pET-32a (+) using specific primers listed in Supplementary Table 3. Recombination plasmids were transformed into *Escherichia coli* BL21 (DE3) pLysS (Promega, Madison, WI, USA). Protein recombination was carried out as reported with some modifications (Xie et al., 2020). Recombinant proteins were induced by adding 500 μM IPTG and cultured at 16°C for 20–24 h. HisTALON Gravity Columns (Takara Bio Inc., Beijing, China) was used to purify His-tagged proteins according to manual. PD-10 columns (GE Healthcare, UK) was used to desalt of His-tagged proteins. Recombinant proteins were monitored by SDS-PAGE and quantitated by BCA kit (FUDE, Hangzhou, China).

### Enzyme assay

Enzymatic activity assay was carried out as reported with some modifications (Ren et al., 2022). Reactions were performed in a total volume of 100 μl mixture containing 0.1 M Tris–HCl buffer (pH 7.5), 1 mM sugar donors (UDP-Glc/UDP-Gal/UDP-Rha), 60 μM sugar acceptors (Q/C), and 1–2 μg recombinant proteins at 30°C for 20 min. Enzyme reactions were stopped by adding 100 μl methanol, and analyzed by HPLC after centrifugation (12,000 rpm for 15 min) as reported (Xie et al., 2020). Enzyme products were detected at 350 nm for flavonol glycosides and at 520 nm for anthocyanins. Enzyme products were analyzed by LC-MS/MS as reported (Ren et al., 2022).

### Site-directed mutagenesis analysis

Mutant proteins were generated by overlapping PCR using primers listed in Supplementary Table 4. Mutant sequences were confirmed by sequencing. Recombinant mutant proteins were monitored by SDS-PAGE. Reaction for site-directed mutagenesis analysis was carried out as mentioned above, and 1–2 μg recombinant mutated proteins were contained in reaction mixture. Relative activity of mutant enzyme was quantified using HPLC.

### Transient expression in *Nicotiana benthamiana*

Transient expression in *N. benthamiana* was performed as reported (Cao et al., 2019). Coding sequences of *MrUFGT* and *MrUGT72B67* were subcloned into pGreenII0029 62-SK (SK) vector. Specific primers were listed in Supplementary Table 5. All recombinant plasmids were electroporated into *Agrobacterium tumefaciens* strain GV3101. Bacteria were resuspended in infiltration bufefr (150 μM acetosyringone, 10 mM MgCl_2_, 10 mM MES, pH 5.6) to OD_600_ of 0.75. Mixtures were prepared according to combination information in Figure 8A. Each combination contained *A. tumefaciens* strain p19. Four-week-old *N. benthamiana* leaves were infiltrated with different combination mixtures. Flavonoid glycosides were analyzed by LC-MS/MS after 5 days infiltration as previous reported (Ren et al., 2022). Data were collected from at least three independent *N. benthamiana* plants.

**FIGURE 1:**
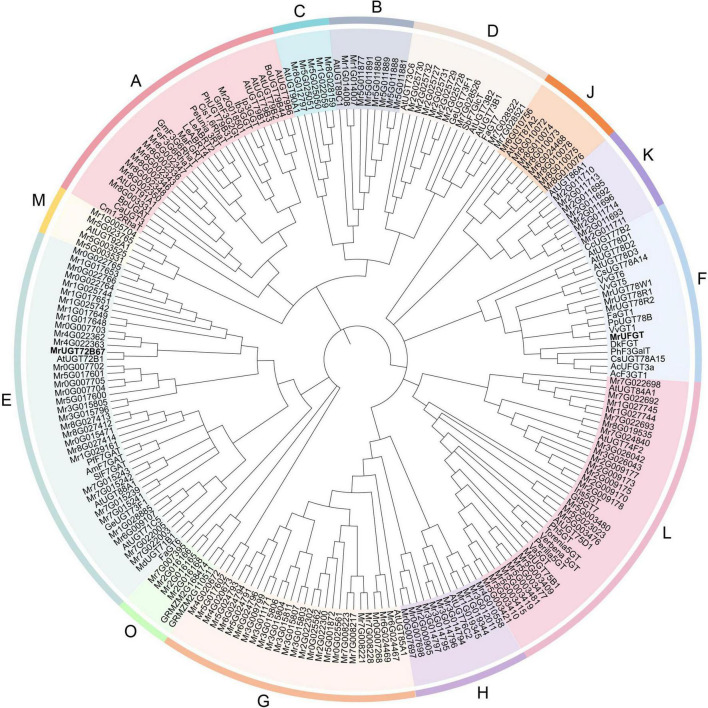
Phylogenetic analysis of *Morella rubra* UDP-glycosyltransferase (UGT) gene family. Phylogenetic tree was constructed by neighbor-joining method. Groups are shown in different colors. Abbreviations of species names are follows: AC, *Aralia cordata*; Am, *Antirrhinum majus*; At, *Arabidopsis thaliana*; Bo, *Brassica oleracea*; Bp, *Bellis perennis*; Ca, *Catharanthus roseus*; Cc, *Crocosmia* × *crocosmiiflora*; Cm, *Citrus maxima*; Cis, *Citrus sinensis*; Cs, *Camellia sinensis*; Dk, *Diospyros kaki*; Fa, *Fragaria* × *ananassa*; Fe, *Fagopyrum esculentum*; Ge, *Glycyrrhiza echinata*; Gm, *Glycine max*; Gt, *Gentiana triflora*; Iris, *Iris hollandica*; Ib, *Ipomoea batatas*; Ip, *Ipomoea nil*; Le, *Lobelia erinus*; Ma, *Morus alba*; Md, *Malus* × *domestica*; Perilla, *Perilla frutescens*; Ph, *Petunia hybrida*; Pf, *Perilla frutescens*; Pp, *Prunus persica*; Sb, *Scutellaria baicalensis*; Sl, *Scutellaria laeteviolacea*; Torenia, *Torenia hybrid*; Va, *Vitis amurensis*; Verbena, *Verbena hybrida*; Vv, *Vitis vinifera*. Accession numbers of UGTs from other species are shown in Supplementary Table 1.

**FIGURE 2:**
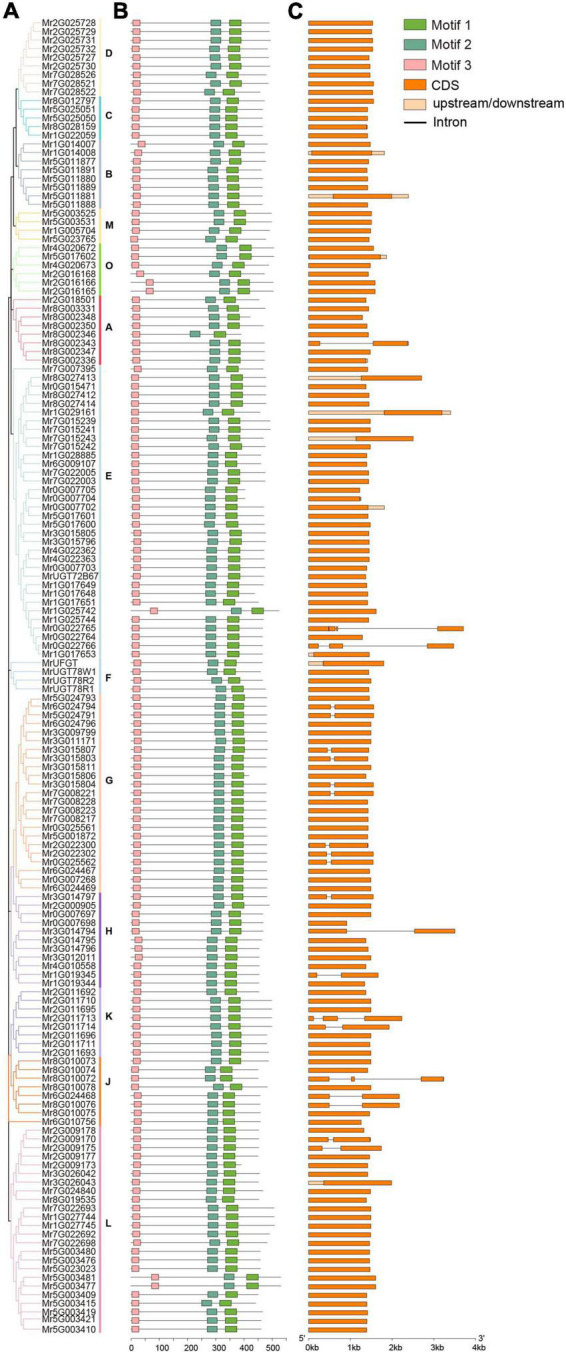
Analysis of phylogenetic relationship **(A)**, conserved motif **(B)**, and gene structure **(C)** of MrUGT gene family. The 14 groups are shown with different colors. Three conserved motifs were shown with different colors. Orange box represented exons and black lines represented introns.

**FIGURE 3:**
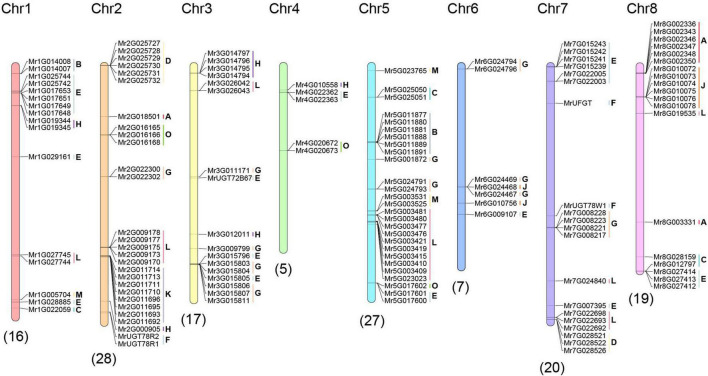
Chromosome distribution of *MrUGT* genes. Numbers of chromosomes were labeled at the top of each chromosome. Numbers of *MrUGT*s on chromosome were labeled at the bottom of each chromosome. Group of *MrUGT*s is labeled next to *MrUGT* accession numbers.

**FIGURE 4:**
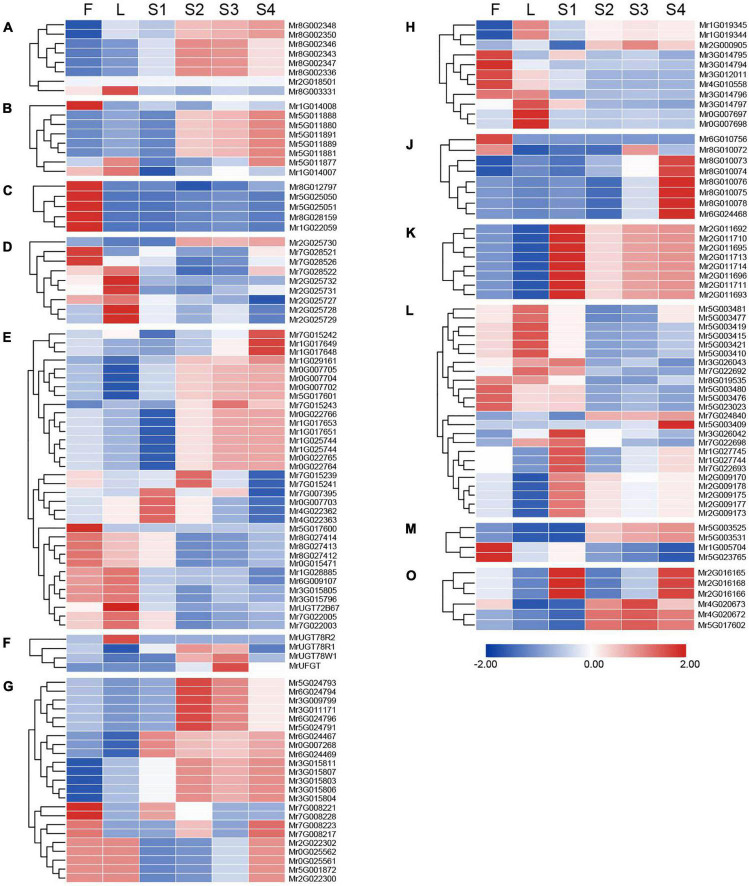
Expression pattern of *MrUGT* genes in different tissues of *Morella rubra*. Expression of *MrUGT* genes in flowers (F), leaves (L), and fruit development (S1–S4) are shown. Color scale represents –2 to 2. **(A–H)**, **(J–M)**, and **(O)** mean different phylogenetic groups of *MrUGT* genes.

**FIGURE 5:**
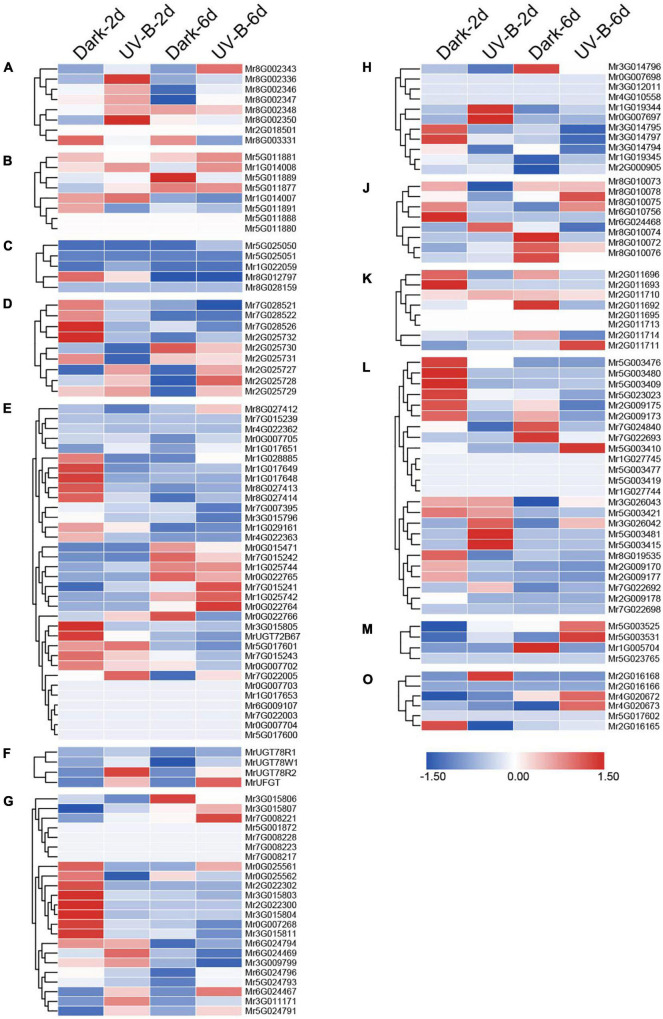
Expression pattern of *MrUGT* genes in response to UV-B irradiation. Color scale represents –1.5 to 1.5. **(A–H)**, **(J–M)**, and **(O)** mean different phylogenetic groups of *MrUGT* genes.

**FIGURE 6:**
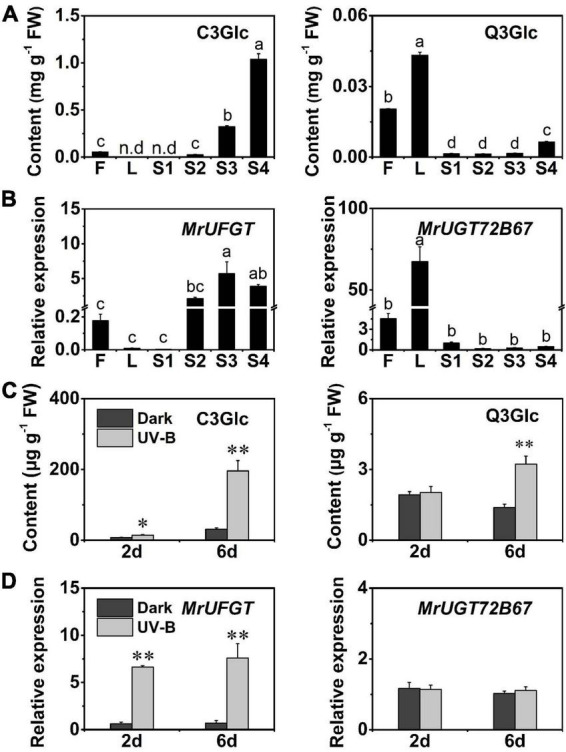
Flavonoid glucosides accumulation and gene expression of *MrUGT*s in different tissues and UV-B-treated fruit. **(A)** Accumulation of cyanidin 3-*O*-glucoside (C3Glc) and quercetin 3-*O*-glucoside (Q3Glc) in flowers (F), leaves (L), and fruit development stages (S1–S4) of *Morella rubra*. **(B)** Gene expression of *MrUFGT* and *MrUGT72B67* in different tissues. Different letters indicate significant difference between different groups (*P* < 0.05). **(C)** Effects of UV-B irradiation on content of C3Glc and Q3Glc in *M. rubra* fruit. **(D)** Gene expression of *MrUFGT* and *MrUGT72B67* in response to UV-B irradiation. Student’s *t*-test is used for statistical analyses between two samples (**P < 0.01, *P < 0.05). All data are presented as the mean ± SE (*n* = 3).

**FIGURE 7:**
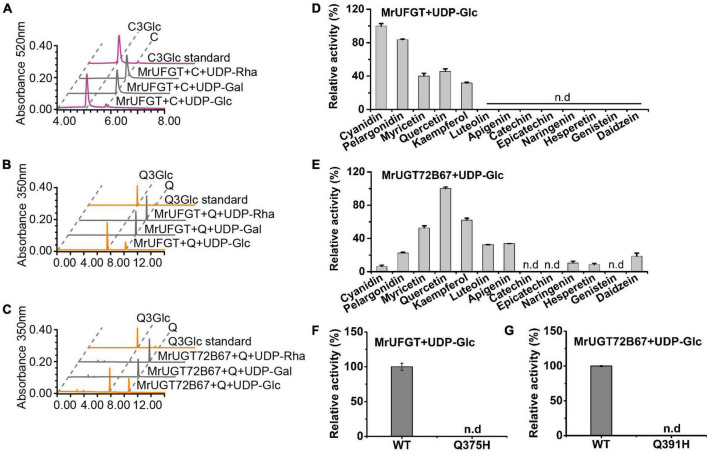
Enzymatic assay of MrUFGT and MrUGT72B67. Enzyme activity analysis of recombinant MrUFGT with cyanidin **(A)** and quercetin **(B)** as sugar acceptors, UDP-glucoside (UDP-Glc), UDP-galactoside (UDP-Gal), and UDP-rhamnoside (UDP-Rha) as sugar donors. **(C)** Enzyme activity analysis of recombinant MrUGT72B67 with quercetin as sugar acceptor, UDP-Glc, UDP-Gal, and UDP-Rha as sugar donors. Relative activities of recombinant MrUFGT with UDP-Glc **(D)** and MrUGT72B67 with UDP-Glc **(E)** toward various flavonoids. Site-directed mutagenesis analysis of MrUFGT **(F)** and MrUGT72B67 **(G)** with quercetin as acceptor and UDP-Glc as sugar donor. Data are presented as mean ± SE (*n* = 3). n.d, not detected.

**FIGURE 8:**
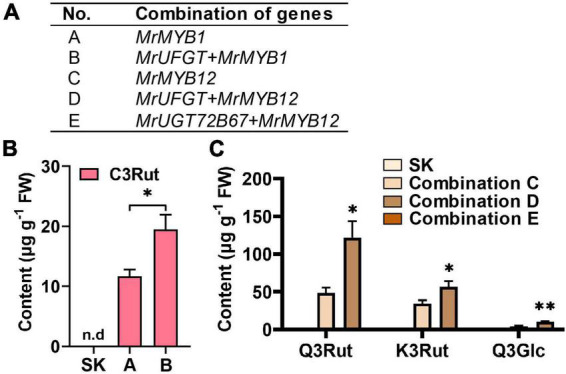
Transient expression of MrUGTs in *Nicotiana benthamiana*. **(A)** Transient expression information of combination mixtures. **(B)** Analysis of anthocyanins in *N. benthamiana* leaves infiltrated with combinations contained MrUFGT. **(C)** Analysis of flavonol glucosides in *N. benthamiana* leaves infiltrated with combinations contained MrUFGT or MrUGT72B67. Leaves infiltrated with empty SK vector were used as control. Products were confirmed by LC-MS/MS. Data are mean ± SE (*n* = 3).

### Statistical analysis

One-way ANOVA followed Tukey test was performed to analyze significant differences among different groups at a significance level of 0.05 using DPS 9.01. Two-tailed Student’s *t*-test was used to analyze two-sample statistical significance. Experimental data were analyzed and presented by Origin 9.0 (Northampton, MA, USA) and GraphPad Prism 9 (San Diego, CA, USA). All experimental data were collected from at least three biological replicates. Error bar was presented as standard error (SE).

## Results

### Identification and phylogenetic analysis of *MrUGT* gene family

To identify UGT gene family in *M. rubra* genome, an HMM profile (PF00201) was used as a query file to find MrUGT proteins. The screening criteria was that the *E*-value < 1. After manual correction of incomplete sequences based on RNA-Seq database, sequences containing more than 350 amino acids were chosen for further analysis. A total of 152 predicted amino acid sequences with conserved PSPG-box were obtained. A phylogenetic tree was constructed with other plant UGTs to investigate functional UGT in *M. rubra*. Results showed that MrUGTs were phylogenetically divided into 13 major groups, i.e., A–H, J–M, and O (Figure 1). Among them, 12 groups (A–H, J–M) were identified in *Arabidopsis* (Ross et al., 2001) and one group (group O) was newly identified (Caputi et al., 2012). Group I and N were absent in *M. rubra* genome (Figure 1).

### Analysis of conserved motif and gene structure of *MrUGT* gene family

To investigate characteristics of *MrUGT* gene family, conserved motifs and intron-exon structure were analyzed. Number of MrUGT proteins was different in each group. Group E contained the largest members in MrUGT gene family, i.e., 34 MrUGT members (22%) (Figure 2). Followed by group L and group G, the MrUGT number was 24 (16%) and 23 (15%), respectively (Figure 2). Groups F and M contained the least MrUGT members, both were four UGT members (Figure 2). Three conserved motifs were predicted in MrUGT family based on MEME analysis. Motif 1 was conserved PSPG-box, and motif 2 and 3 were conserved in all MrUGT proteins (Figure 2 and Supplementary Figure 1). This indicating that UGT also has other conserved motif in addition to PSPG-box.

Intron-exon structure was investigated to understand gene function and evolutionary relationships within *MrUGT* gene family. Results showed that 22 *MrUGT* members contained introns, accounting for about 15% (Figure 2). In terms of intron numbers, 18 *MrUGT*s contained one intron, three *MrUGT*s had two introns, and one *MrUGT* had three introns (Figure 2). For *UGT* groups, the largest number of *UGT*s with introns was observed in group G, and that was nine members. Followed by group H and J, both groups had three *UGT*s with introns (Figure 2). Most of *MrUGT*s does not had introns, and gene structure was relatively conservative.

### Chromosome distribution and synteny analysis of *MrUGT* gene family

To investigate the distribution of *MrUGT* genes, genomic positions of each *MrUGT* were marked on chromosomes (Figure 3). A total of 139 *MrUGT* genes were marked on eight chromosomes of *M. rubra* and 13 *MrUGT* genes located on unmapped scaffolds (Figure 3 and Supplementary Table 6). There were largest *MrUGT* numbers (28) located on chromosome 2, followed by 27 *MrUGTs* on chromosome 5 and 20 *MrUGTs* on chromosome 7. Only five *MrUGT* genes located on chromosome 4. For the largest MrUGT group (Group E), eight members were distributed on chromosome 1, three members were distributed on chromosome 3, two members were distributed on chromosome 4, two members were distributed on chromosome 5, one member was distributed on chromosome 6, seven members were distributed on chromosome 7, three members were distributed on chromosome 8, and eight members were distributed on unmapped scaffolds (Figure 3 and Supplementary Table 6).

Gene duplication was one of driven forces for gene family expansion (Qiao et al., 2019). Four gene duplication modes were identified in *MrUGT* gene family based on method reported by Qiao et al. (2019), including whole-genome duplication (WGD), dispersed duplication (DSD), tandem duplication (TD), and proximal duplication (PD). A total of 29 TD events were observed in MrUGT gene family, followed by 28 PD events. Only eight DSD events and three WGD events were observed in *MrUGT* gene family (Supplementary Table 7). Group L contained the largest number of gene duplication events, and it was 14. Followed by groups E and G, number of gene duplication events was nine and eight, respectively (Supplementary Table 7).

To further explore evolutionary relationships of *MrUGT*, syntenic maps were constructed between *M. rubra* and three Rosid species, including *Arabidopsis*, *J. regia*, and *P. persica* (Supplementary Figure 2). A total of 22, 41, and 40 homologous *UGT* gene pairs were identified between *M. rubra* and *Arabidopsis*, *J. regia*, and *P. persica*. It indicated that *M. rubra* has a closer evolutionary relationship with *J. regia* and *P. persica*, which was consistent with the study of *M. rubra* genome (Jia et al., 2019).

### Tissue and temporal expression pattern of *MrUGT* genes in *Morella rubra*

RNA-seq was performed to analyze expression pattern of *MrUGT* genes in flowers, leaves, and fruit development stages of ‘BQ’ cultivar. A total of 29 *MrUGT* genes showed the highest expression level in flowers (Figure 4). All *UGT* members in group C exhibited highest expression level in flowers (Figure 4). The other *MrUGT* genes expressed highest in flowers were mainly from group E, G, and H (Figure 4). A total of 30 *MrUGT* genes showed the highest expression level in leaves. More than half of members in group D and H were mainly expressed in leaves (Figure 4). Notably, a total of 99 *MrUGT* members were mainly expressed in fruit, accounting for 65% of total *MrUGT*. Among them, 31 *MrUGT* members had the highest expression level in S1 stage, 23 *MrUGT*s showed the highest expression level in S2 stage, 11 *MrUGT*s showed the highest expression level in S3 stage, and 34 *MrUGT*s showed the highest expression level in S4 stage (Figure 4). 22 members of the largest group (group E) showed the highest expression level in fruit (Figure 4). All members of group K and group O had the highest expression level in fruit (Figure 4). Expression pattern analysis indicated that *MrUGT*s played important roles in metabolic pathways related to fruit development and ripening.

### Expression pattern of *MrUGT* genes in response to ultraviolet-B irradiation

UV-B stress is an efficient treatment for induction of flavonoid glycosides accumulation in plants (Kolb et al., 2001; Stracke et al., 2010; Henry-Kirk et al., 2018; Xie et al., 2022). Therefore, we carried out UV-B treatment for investigation and identification of MrUGTs involved in flavonoid glucosylation (Figure 5). Based on transcriptomic analysis, gene expression of 13 *MrUGT* genes were significantly induced (log_2_FC >1, *p* < 0.05) by UV-B treatment. Among them, seven *MrUGT* genes were significantly induced after 2 days UV-B treatment, and ten *MrUGT* genes were significantly induced after 6 days UV-B treatment (Supplementary Table 8). Four *MrUGT* genes were significantly induced by UV-B treatment after both 2 and 6 days.

Based on current knowledge, UGTs in group F were closely related to flavonoid 3-*O*-glycoside formation. Among the UV-B induced MrUGTs, only four members belong to group F, i.e., MrUGT78R1, MrUGT78R2, MrUGT78W1, and MrUFGT (Supplementary Table 8). Recently, MrUGT78R1 and MrUGT78R2 were identified as UDP-rhamnosyltransferases while MrUGT78W1 was identified as UDP-galactosyltransferase involved in flavonol glycosylation in *M. rubra* by our group (Ren et al., 2022). Therefore, MrUFGT was chosen as one of potential candidate UGTs for flavonoid glucosylation.

### Identification of MrUGTs related to flavonoid glucoside accumulation

Flavonoid glucoside profiles in different tissues of *M. rubra* were analyzed by HPLC. Flavonoid glucosides accumulation exhibited tissue specificity in *M. rubra*. C3Glc was mainly accumulated in mature fruit (S4) and flowers (Figure 6A). While Q3Glc was mainly accumulated in leaves and flowers (Figure 6A).

Correlation analysis between C3Glc content and expression of *MrUGT*s in different tissues was performed. A total of 12 *MrUGT* genes showed high correlation coefficient (*r* > 0.8) with C3Glc content, where only MrUFGT belongs to group F of UGT family (Supplementary Figure 3A). Similarly, correlation analysis between Q3Glc content and expression of *MrUGT*s in different tissues was performed. A total of 9 *MrUGT* genes showed high correlation coefficient (*r* > 0.8) with Q3Glc content (Supplementary Figure 3B). However, none of these 9 MrUGTs belongs to group F of UGT family. *MrUGT72B67* in group E showed the highest expression in leaves and flowers, and was thus chosen for recombinant protein expression and enzymatic assay.

Gene expression of *MrUFGT* and *MrUGT72B67* was confirmed by RT-qPCR (Figure 6B). Results showed that *MrUFGT* was mainly expressed in fruit and flowers, and increased during fruit development (Figure 6B), which was consistent with C3Glc accumulation pattern. While *MrUGT72B67* was mainly expressed in leaves and flowers (Figure 6B), which was consistent with Q3Glc accumulation pattern in leaves and flowers. UV-B treatment could significantly induce C3Glc and Q3Glc accumulation in ‘Biqi’ fruit (Figure 6C). And gene expression of *MrUFGT* was significantly induced by UV-B, while *MrUGT72B67* were not (Figure 6D).

### Enzymatic assays of recombinant MrUFGT and MrUGT72B67

*MrUFGT* and *MrUGT72B67* were isolated from cDNA libraries of ‘Biqi’ cultivar. ORFs of *MrUFGT* and *MrUGT72B67* were 1,389 and 1,422 bp, which encoded predicted proteins composed of 462 and 473 amino acids, respectively. Phylogenetic analysis indicated that MrUFGT and MrUGT72B67 exhibited the highest homology with VvGT1 and AtUGT72B1, respectively (Supplementary Figure 4). Sequence alignment analysis showed that PSPG-box of MrUFGT and MrUGT72B67 was conserved and closed to C-terminal (Supplementary Figure 5). Recombinant proteins of MrUFGT and MrUGT72B67 were verified by SDS-PAGE (Supplementary Figure 6). Enzymatic assays were performed to verify functions of MrUFGT and MrUGT72B67. Results showed that MrUFGT could only transfer UDP-Glc to anthocyanidin or flavonol aglycones. Product peaks with *m/z* 448 and *m/z* 463 tentatively identified as C3Glc and Q3Glc based on fragmentation information (Figures 7A,B and Supplementary Figure 7). MrUFGT could not transfer UDP-Rha or UDP-Gal to anthocyanidins or flavonol aglycones such as C or Q (Figures 7A,B). MrUGT72B67 could only transfer UDP-Glc to flavonol aglycones, resulting in formation of peak with *m/z* 463 which was tentatively identified as Q3Glc (Figure 7C and Supplementary Figure 7). MrUGT72B67 could not transfer UDP-Rha or UDP-Gal to flavonol aglycone such as Q (Figure 7C).

Enzyme activity of MrUFGT and MrUGT72B67 for different flavonoid aglycones were also investigated. For MrUFGT, C was the best substrate since MrUFGT showed the highest activity toward it. For different substrates (flavonoid aglycones), relative enzyme activities of MrUFGT were calculated by comparison of enzyme activity toward each substrate with that of C. As a result, MrUFGT showed relative lower activity for flavonol aglycones (M, Q, and K) compared to anthocyanidin aglycones (Figure 7D). MrUFGT did not exhibit glucosyl transfer activity toward naringenin, hesperetin, epicatechin, catechin, luteolin, apigenin, genistein, and daidzein (Figure 7D). For MrUGT72B67, Q was the best substrate since MrUGT72B67 showed the highest activity toward it. For different substrates (flavonoid aglycones), relative enzyme activities of MrUGT72B67 were calculated by comparison of enzyme activity toward each substrate with that of Q. As a result, MrUGT72B67 showed relative lower activity for C, P, naringenin, hesperetin, luteolin, apigenin, and daidzein compared to Q (Figure 7E). It indicated that MrUGT72B67 displayed a relatively broad substrate preference toward flavonoid.

To explore the role of last amino acid residue in PSPG-box for glucosyl transfer activity of MrUGTs, site-directed mutagenesis was carried out. Two mutant proteins (Q375H of MrUFGT and Q391H of MrUGT72B67) were generated by overlapping PCR (Supplementary Figure 8). Q375H mutation and Q391H mutation completely lost the glucosyltransferase activity of MrUFGT and MrUGT72B67, respectively (Figures 7F,G). No mutations resulted in additional galactosyltransferase or rhamnosyltransferase activity (Supplementary Figure 9).

### Transient expression of *MrUGTs* in *Nicotiana benthamiana*

To validate functions of *MrUFGT* and *MrUGT72B67 in vivo*, transient expression was carried out in *N. benthamiana* plants. Anthocyanin- and flavonol-specific transcription factors *MrMYB1* (Niu et al., 2010) and *MrMYB12* (Cao et al., 2021) were introduced to transient expression system to enhance substrates level of UGT according to reported (Irmisch et al., 2019; Figure 8A).

*Nicotiana benthamiana* leaves accumulated cyanidin 3-*O*-rutinoside (*m/z* 593 with MS^2^ fragmentation at *m/z* 285, C3Rut) when only expressed with MrMYB1 (combination A) (Figure 8B and Supplementary Figure 10). And level of C3Rut was significantly enhanced when *MrUFGT* was added (combination B) (Figure 8B and Supplementary Figure 10). And flavonol glucoside derivatives, i.e., Q3Rut (*m/z* 609 with MS^2^ fragmentation at *m/z* 300), K3Rut (*m/z* 593 with MS^2^ fragmentation at *m/z* 258), and Q3Glc (*m/z* 463 with MS^2^ fragmentation at *m/z* 300), were significantly accumulated in *N. benthamiana* leaves infiltrated with *MrUFGT* (combination D) compared to infiltrated *MrMYB12* only (combination C) (Figure 8C and Supplementary Figure 10). Like MrUFGT, flavonol glucoside derivatives (Q3Rut, K3Rut, and Q3Glc) were significantly accumulated in *N. benthamiana* leaves with addition of *MrUGT72B67* (combination E) compared to expressed *MrMYB12* only (combination C) (Figure 8C and Supplementary Figure 10). Control leaves did not accumulate anthocyanins or flavonol glycosides at detectable level (Figure 8 and Supplementary Figure 10). These results tested the functional glucosyltransferase activity of MrUFGT and MrUGT72B67.

## Discussion

### UDP-glycosyltransferase gene family contribute to diversity of secondary metabolites

*Morella rubra* is rich in flavonoid glycosides, and different tissues have been used historically as folk medicines. Here we reported the genome-wide analysis of *UGT* gene family and identified two MrUGTs involved in the accumulation of flavonoid glucosides.

The first plant reported *UGT* gene family was *Arabidopsis* and 107 *UGT* genes was identified in genome (Li et al., 2014). In the present study, a total of 152 *UGT* genes were identified in *M. rubra* genome. Number of *MrUGT* gene was little difference compared to other species, examples include 241 *UGT*s in *M. domestica* (Caputi et al., 2012), 147 *UGT*s in *Z. mays* (Li et al., 2014), and 168 *UGT*s in *P. persica* (Wu et al., 2017). It indicated that *MrUGT* gene family did not exhibit significant expansion compared with other plants, which may be related to the lack of recent genome-wide duplication events in *M. rubra* (Jia et al., 2019). Based on phylogenetic relationship, UGTs in *Arabidopsis* were clustered into 14 groups (A-N) (Li et al., 2001). And four new groups, i.e., O, P, Q, and R, were discovered subsequently in *M. domestica* (Caputi et al., 2012), *Z. mays* (Li et al., 2014), and *C. sinensis* (Cui et al., 2016). *MrUGT* gene family contained 13 groups (Figure 1), including 12 groups discovered in *Arabidopsis* and one newly discovered group O, and with absent of group I, N, P, Q, and R compared to 18 groups (A-R) reported in plants. This indicated that gene loss events occurred during *UGT* gene family expansion in *M. rubra*.

UDP-glycosyltransferases prefer to be clustered by regiospecificity rather than species and sugar donor specificity (Yonekura-Sakakibara and Hanada, 2011; Hsu et al., 2017). Therefore, it is considered that sugar donor specificity differentiation was later than divergence of regiospecificity (Hsu et al., 2017). This makes it possible to predict functional UGTs by phylogenetic analysis. For example, UGT members in group A were considered to be related to biosynthesis of flavonoid disaccharides, and examples include Cis1,6RhaT (Frydman et al., 2004), Cm1,2RhaT (Frydman et al., 2013), and PpUGT79AK6 (Xie et al., 2022). UGT members in group O usually exhibited activity toward plant hormone zeatin. For example, PvZOX1 from *Phaseolus vulgaris* was identified as a zeatin *O*-xylosyltransferase involved in *O*-xylosylzeatin formation (Martin et al., 1999). And cisZOG1 from *Z. mays* was identified as a glucosyltransferase specific to *cis*-zeatin (Martin et al., 2001). Phylogenetic homology analysis of UGTs would facilitate the discovery of more functional UGTs in plants with specialized metabolites.

Gene duplication is important for gene family expansion, and results in gene clusters on chromosomes. Gene duplication events include five modes according to Qiao et al. (2019), i.e., WGD, TD, DSD, PD, and transposed duplication (TRD). Four modes except TRD were found in *MrUGT* family. About 19% and 18% *MrUGT* genes occurred TD and PD events, respectively, indicating that both TD and PD were ongoing processes throughout evolutionary of *MrUGT* gene family. In *Broussonetia papyrifera*, TD was primary driving force for expansion of *BpUGT* gene family (Wang et al., 2021). In higher plants, it was found that groups A, D, E, G, and L expanded more than other groups during plant evolution (Caputi et al., 2012). In *M. rubra*, groups E, G, and L were expanded significantly compared with other groups, which was mainly related to gene duplication events in these groups.

### MrUFGT and MrUGT72B67 involved in flavonoid glucosylation

Various anthocyanins and flavonol glycosides are of interest to researchers because of their importance in plant physiology and human health. To date, many plant UGTs involved in biosynthesis of anthocyanins and flavonol glycosides are reported. Flavonoid 3-*O*-glycosyltransferase (UFGT) *bronze1* from maize was the first identified UGT in plant that only used UDP-Glc for the biosynthesis of anthocyanins, which were important for pigment accumulation in maize (Dooner and Nelson, 1977). In grape, VvGT1 was a flavonoid 3-*O*-glucosyltransferase that catalyzed anthocyanins formation during grape fruit ripening (Ford et al., 1998). In model plant *Arabidopsis*, AtUGT78D2 was identified as flavonoid 3-*O*-glucosyltransferase by enzymatic activity analysis and T-DNA-inserted mutants (Tohge et al., 2005). Recently, PpUGT78T3 was identified as UDP-glucosyltransferase involved in regulation of flavonol glucosides in response to UV-B (Xie et al., 2022).

In this work, both transcriptomic data and phylogenetic homology of UGT subgroups together with their correlation with flavonoid accumulation patterns in different tissues or under UV-B treatment were comprehensively analyzed for screen of candidate UGTs involved in flavonoid glucosides accumulation. MrUFGT was mainly screened based on phylogenetic homology analysis with group F and correlation relationship between flavonoid glucosides contents and its expression, while MrUGT72B67 was screened based on tissue specific accumulation of flavonoid glucosides and its transcriptomic analysis. Here we demonstrated that MrUFGT was involved in C3Glc accumulation by *in vitro* and *in vivo* experimental data. In addition, MrUFGT exhibited activity toward Q resulting in Q3Glc formation. However, Q3Glc accumulation pattern in flowers and leaves was not correlation with gene expression pattern of *MrUFGT*. This indicating that there might be another UGT member involved in Q3Glc accumulation in flowers and leaves. MrUGT72B67 in group E was found to be involved in Q3Glc accumulation in leaves and flowers by gene expression analysis as well as *in vitro* and *in vivo* data. Taken together the results of C3Glc and Q3Glc induced by UV-B treatment (Figure 6C), we concluded that MrUFGT mainly involved in accumulation of C3Glc and Q3Glc in fruit, while MrUGT72B67 mainly involved in accumulation of Q3Glc in flowers and leaves.

UDP-glycosyltransferase members in group F were closely related to flavonoid 3-*O*-glycoside formation (Ono et al., 2010; Cheng et al., 2014; Cui et al., 2016; Xie et al., 2022). For example, VvGT5 and VvGT6 in group F from *Vitis vinifera* were identified as flavonol 3-*O*-glucuronosyltransferase and bifunctional flavonol 3-*O*-glucosyltransferase/galactosyltransferase in grapevines (Ono et al., 2010). In *C. sinensis*, CsUGT78A14 and CsUGT78A15 in group F were reported to be responsible for biosynthesis of flavonol 3-*O*-glucosides and flavonol 3-*O*-galactosides, respectively (Cui et al., 2016). PpUGT78A2 in group F was identified as a flavonoid 3-*O*-glycosyltransferase involved in different glycosylation of anthocyanin and flavonol in *P. persica* (Cheng et al., 2014; Xie et al., 2022). And in *M. rubra*, four UGT members in group F, i.e., MrUGT78R1, MrUGT78R2, MrUGT78W1, and MrUFGT in the present study, were identified as flavonoid 3-*O*-glycosyltransferases involved in accumulation of diverse flavonoid glycosides (Ren et al., 2022).

UDP-glycosyltransferase members in group E have been reported with diverse functions in many plants. In *Arabidopsis*, AtUGT72B1 was identified as a bifunctional *O-*glucosyltransferase and *N-*glucosyltransferase involved in metabolism of pollutant 3,4-dichloroaniline (Loutre et al., 2003), and it was also involved in glucose conjugation of monolignols, which play an important role in cell wall lignification in *Arabidopsis* (Lin et al., 2016). In *Lotus japonicus*, three UGTs from group E, i.e., UGT72AD1, UGT72AH1, and UGT72Z2, were identified as glucosyltransferases involved in flavonol glucoside/rhamnoside biosynthesis in *L. japonicus* seeds (Yin et al., 2017).

### Key amino acids in glucosyltransferases

Crystal structure analysis of UGTs have showed that last amino acid residue in PSPG-box was critical for glycosyl transfer activity of UGT (Shao et al., 2005; Offen et al., 2006; Osmani et al., 2009). Last amino acid residue of PSPG-box in UDP-glucosyltransferases usually was glutamine (Gln), such as observed in UGT78D2 from *Arabidopsis* (Tohge et al., 2005), CsUGT78A14 from tea plant (Cui et al., 2016), and FaGT6 and FaGT7 from strawberry (Griesser et al., 2008). Some site-directed mutagenesis indicated the important role of Gln as last amino acid residue in PSPG-box. For example, by replacing Gln382 with His in UBGT from *Scutellaria baicalensis*, UBGT exhibited remarkable decrease in glucosyltransferase activity (Kubo et al., 2004). In VvGT1, Q375H mutation completely abolished glucosyl transfer activity, and did not improve galactosyl transfer activity (Offen et al., 2006). Q378H substitution for CsUGT78A14 resulted in glucosyl transfer activity markedly reduced, which indicated that Gln was important for flavonoid 3-*O*-glucosyltransferase activity (Cui et al., 2016).

In the present study, last amino acid residues in PSPG-box were both Gln in MrUFGT (Gln375) and MrUGT72B67 (Gln391). To investigate whether last amino acid residue in PSPG-box was important for glucosyl transfer activity, site-directed mutagenesis of Q375H mutation for MrUFGT and Q391H mutation for MrUGT72B67 were analyzed by enzymatic assay. Results showed that both mutation of Q375H for MrUFGT and Q391H for MrUGT72B67 abolished glucosyl transfer activity. It indicated that Gln as last amino acid residue in PSPG-box were critical for glucosyl transfer activity for MrUFGT and MrUGT72B67.

## Conclusion

In the present study, genome-wide analysis was performed for *UGT* gene family in *M. rubra*, including polygenetic information, chromosomal distribution, gene duplication mode, and expression pattern. A total of 152 *UGT* family members were identified in *M. rubra* genome and clustered into 13 groups based on polygenetic analysis. 139 *MrUGT* genes marked on eight chromosomes and 13 *MrUGT* genes located on unmapped scaffolds. Gene duplication analysis indicated that both tandem and proximal duplication were major drivers for *MrUGT* gene family expansion. Expression analysis indicated *MrUGT*s played important roles during fruit development and ripening. MrUFGT (MrUGT78A26) and MrUGT72B67 were identified as UDP-glucosyltransferases by *in vitro* and *in vivo* experiment which were involved in C3Glc and Q3Glc accumulation in different tissues of *M. rubra*. In addition, Gln375 and Gln391 were identified as important amino acids for glucosyltransferase activity of MrUFGT and MrUGT72B67, respectively.

## Data availability statement

The original contributions presented in this study are publicly available. This data can be found here: NCBI, SRP386597 and SRP310482.

## Author contributions

XL and CR designed the project and drafted the manuscript. CR carried out analyses and experiments with the help of YC, MX, YG, JL, and LX. CS, CX, and KC provided supports to the *M. rubra* project. All authors contributed to the article and approved the submitted version.

## Footnotes

1
http://www.cazy.org

2
http://pfam.xfam.org

3
https://www.ncbi.nlm.nih.gov/Structure/cdd/wrpsb.cgi

4
http://weblogo.berkeley.edu/logo.cgi

5
http://gsds.gao-lab.org/index.php
