# Supplementary material for: Genome-wide analysis of UDP-glycosyltransferase gene family and identification of members involved in flavonoid glucosylation in Chinese bayberry (Morella rubra)
Source: Front Plant Sci. 2022 Sep 26;13:998985. doi: 10.3389/fpls.2022.998985 (PMC9549340; doi:10.3389/fpls.2022.998985)
Supplement: Supplementary file 1 [file Data_Sheet_1.DOCX]

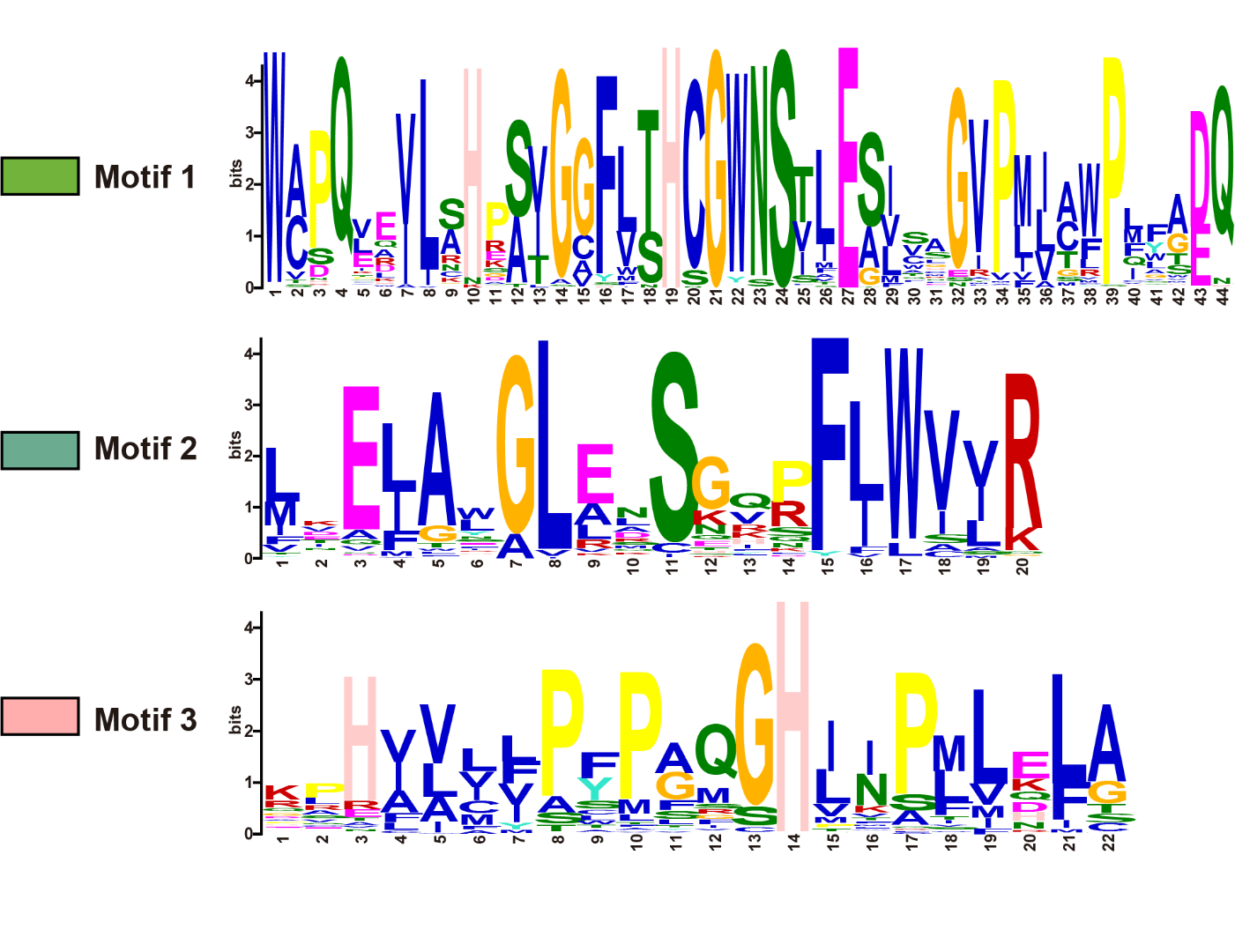


**Figure S1.** Three motif logos identified in MrUGT proteins.


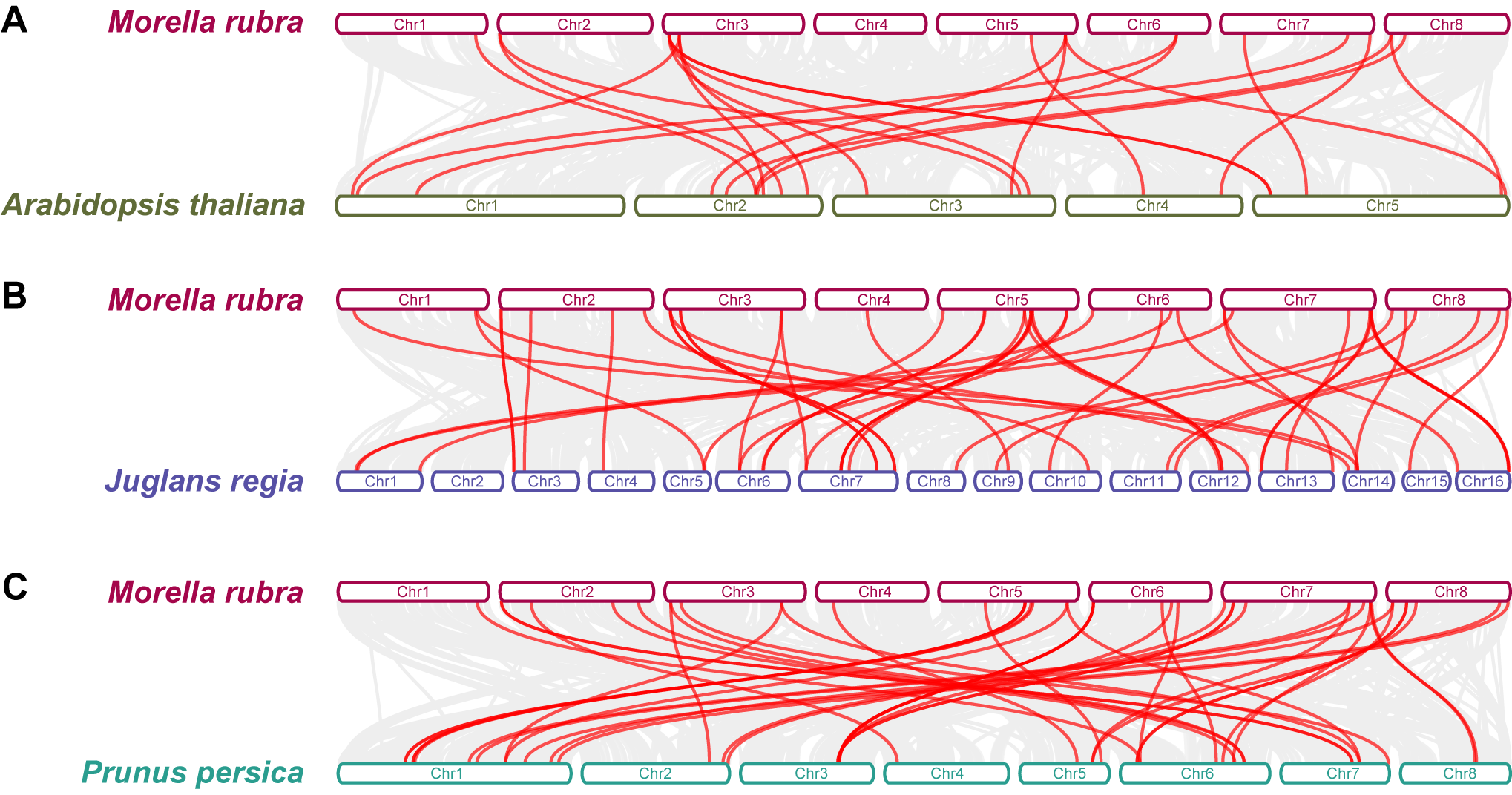


**Figure S2.** Syntenic analysis of *UGT* genes between *M. rubra* and other three representative plant species in Rosids, including *Arabidopsis thaliana* (A), *Juglans regia* (B), and *Prunus persica* (C).


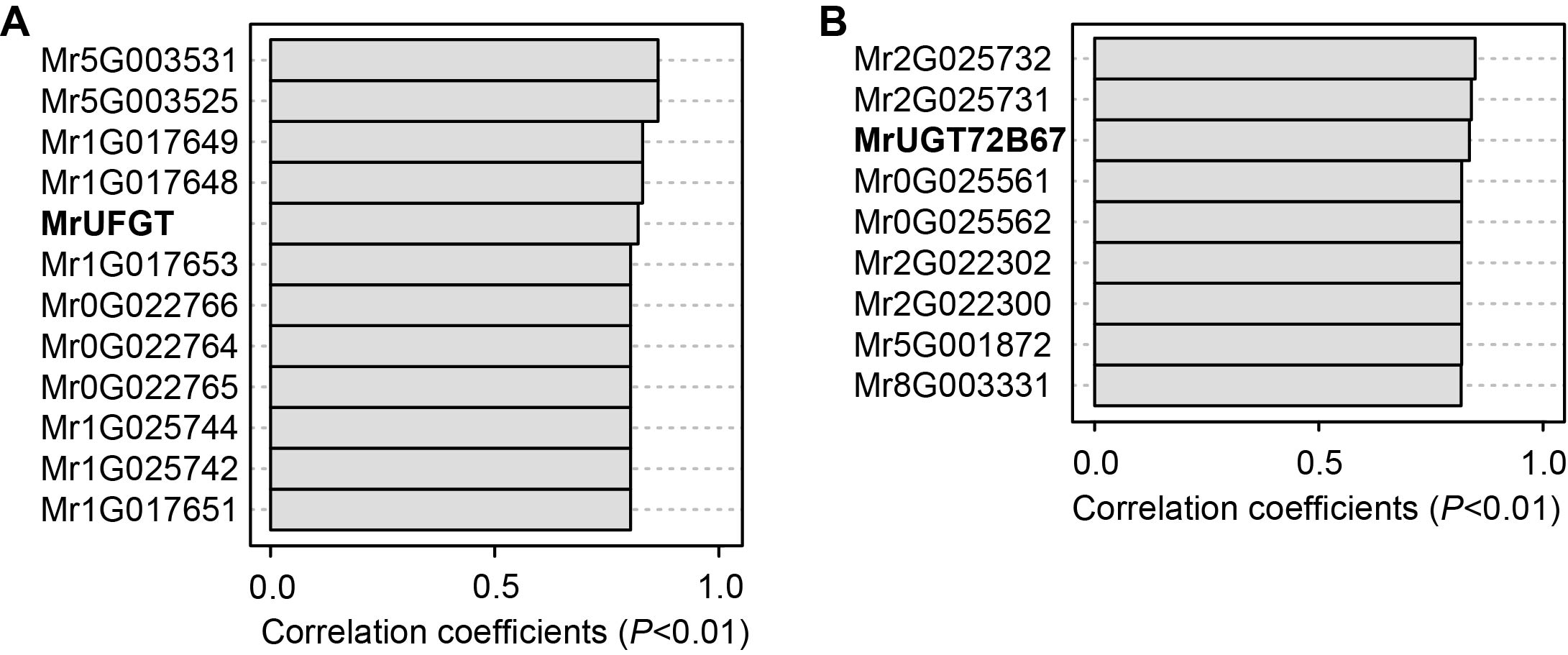


**Figure S3.** Correlation analysis of expression of MrUGTs with accumulation of C3Glc (A) and Q3Glc (B). MrUGTs with correlation coefficient *r* higher than 0.8 were shown for C3Glc and Q3Glc, respectively.


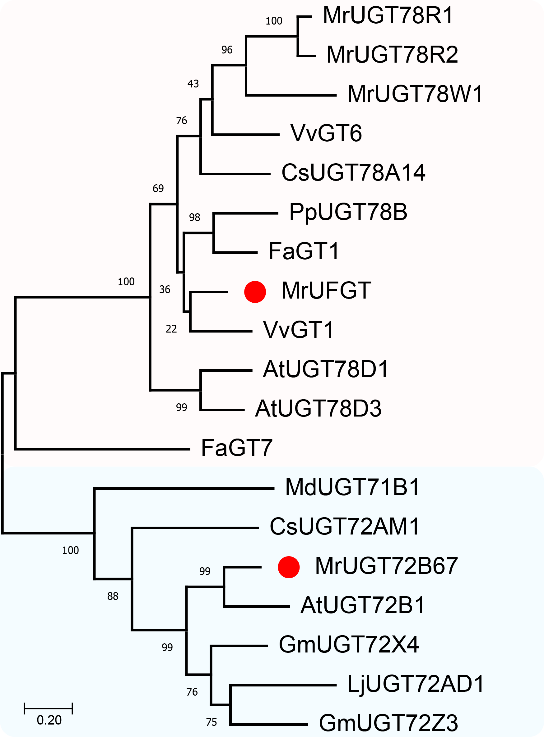


**Figure S4.** Phylogenetic analysis of MrUFGT and MrUGT72B67.


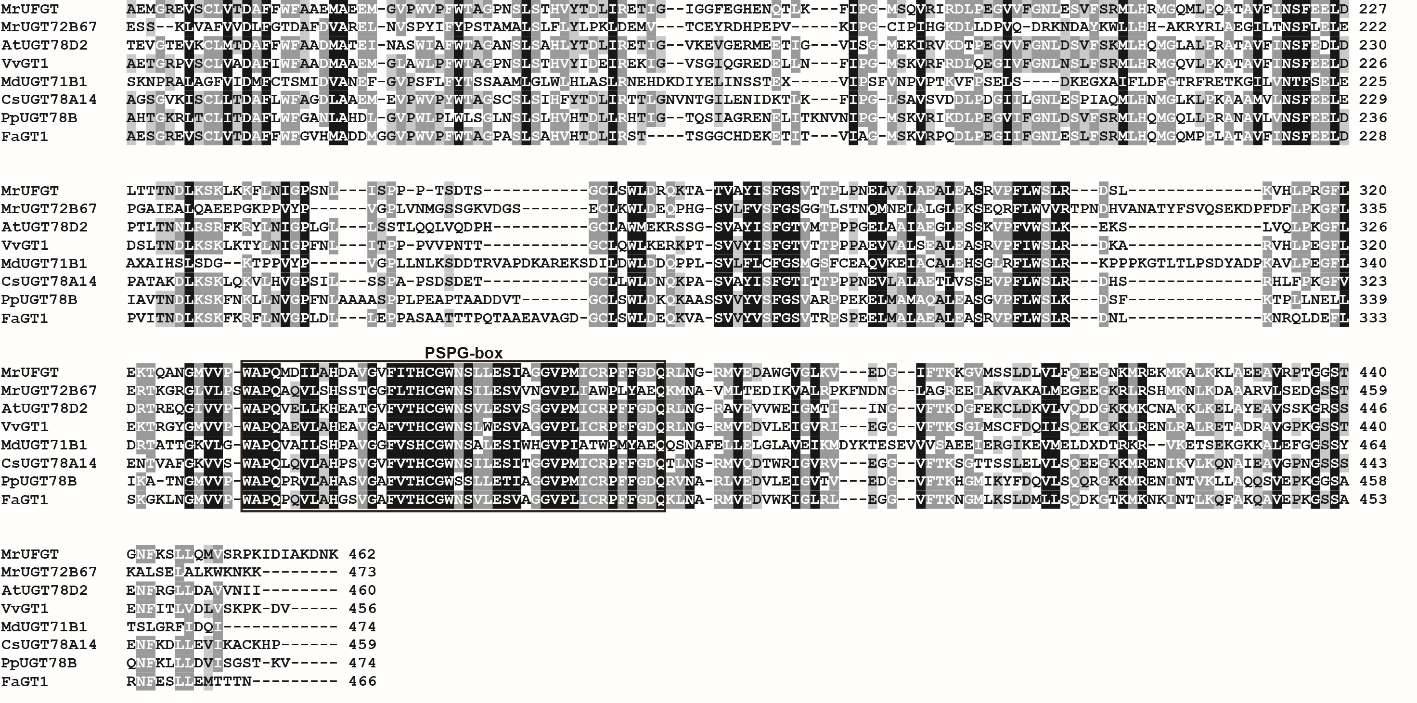


**Figure S5.** Multiple sequence alignment of MrUFGT and MrUGT72B67 with other UDP-glucosyltransferases.


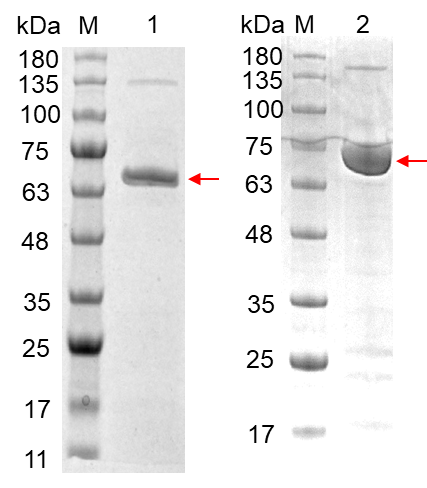


**Figure S6.** SDS-PAGE analysis of the His-tagged MrUFGT (Lane 1) and MrUGT72B67 (Lane 2) proteins. M, protein molecular weight marker. Red arrows indicate recombinant proteins.

**
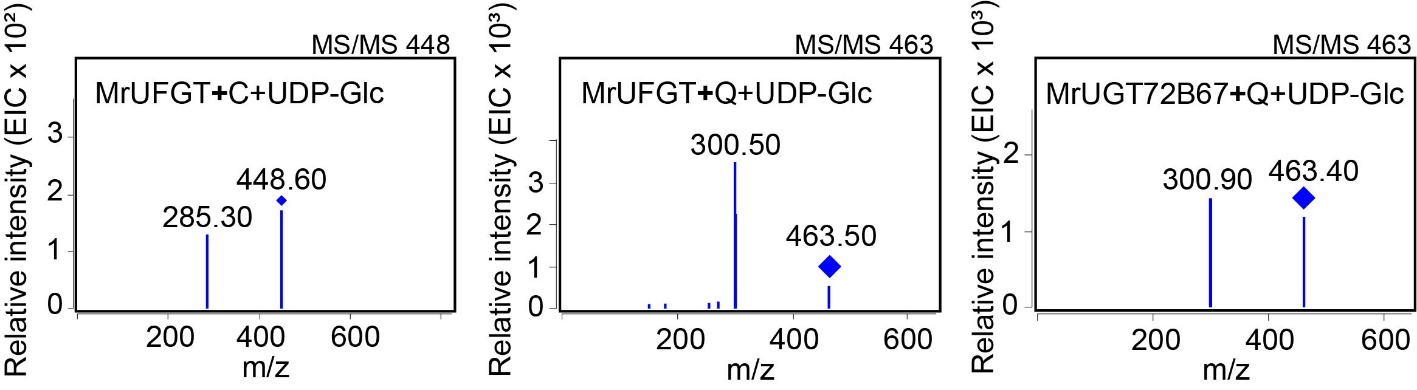
**

**Figure S7.** LC-MS/MS analysis of the products generated by MrUFGT and MrUGT72B67.

**
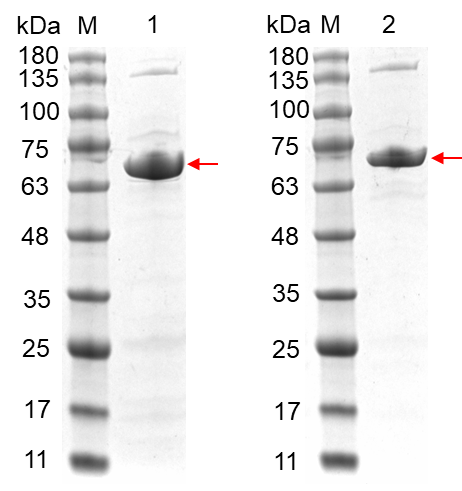
**

**Figure S8.** SDS-PAGE analysis of mutated proteins. Lane 1, Q375H mutation of MrUFGT; Lane 2, Q391H mutation of MrUGT72B67; M, protein molecular weight marker. Red arrows indicate recombinant proteins.

**
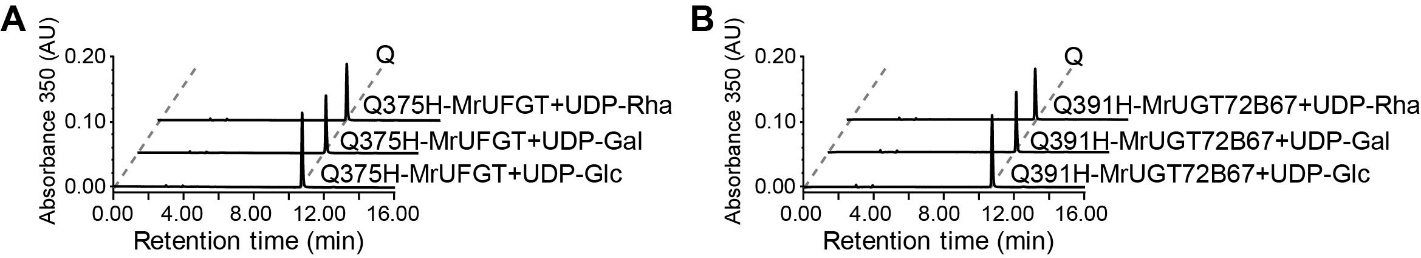
**

**Figure S9.** HPLC analysis of enzyme activity of mutated proteins of MrUFGT (A) and MrUGT72B67 (B) with quercetin as sugar acceptor.

**
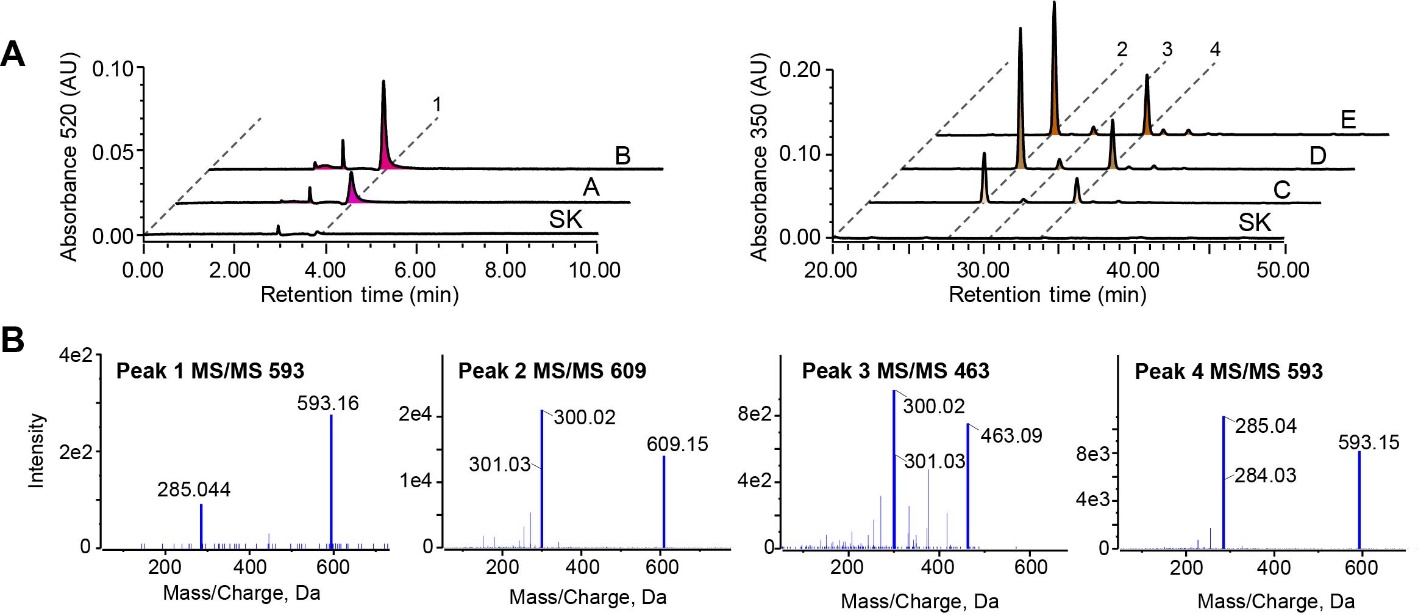
**

**Figure S10.** Identification of products produced by transient expression of MrUGTs in *Nicotiana benthamiana*.

A, HPLC chromatograms of *N. benthamiana* leaves infiltrated with gene combinations. B, Fragmentation information of flavonoid glycosides accumulated in *N. benthamiana* leaves.

Peak 1, cyanidin 3-*O*- rutinoside (C3Rut); Peak 2, quercetin 3-*O*-rutinoside (Q3Rut); Peak 3, quercetin 3-*O*-glucoside (Q3Glc); Peak 4, kaempferol 3-*O*-rutinoside (K3Rut).

**Table S1.** Genbank accession numbers of identified UGTs in phylogenetic tree.

| **Gene** | **Accession number** | **Species** |
| --- | --- | --- |
| AcF3GT1 | GU079683 | *Actinidia chinensis* |
| ACGT | AB103471 | *Aralia cordata* |
| AcUGT3a | Achn209671 | *Actinidia chinensis* |
| AmF7GAT | AB362988 | *Antirrhinum majus* |
| AtUGT71C5 | At1g07240 | *Arabidopsis thaliana* |
| AtUGT72B1 | At4g01070 | *Arabidopsis thaliana* |
| AtUGT73B1 | At4g34138 | *Arabidopsis thaliana* |
| AtUGT73B2 | At4g34135 | *Arabidopsis thaliana* |
| AtUGT73C6 | At2g36790 | *Arabidopsis thaliana* |
| AtUGT74F2 | At2g43820 | *Arabidopsis thaliana* |
| AtUGT75C1 | At4g14090 | *Arabidopsis thaliana* |
| AtUGT75D1 | At4g15550 | *Arabidopsis thaliana* |
| AtUGT76C2 | At5g05860 | *Arabidopsis thaliana* |
| AtUGT78D1 | At1g30530 | *Arabidopsis thaliana* |
| AtUGT78D2 | At5g17050 | *Arabidopsis thaliana* |
| AtUGT78D3 | At5g17030 | *Arabidopsis thaliana* |
| AtUGT79B1 | At5g54060 | *Arabidopsis thaliana* |
| AtUGT79B2 | At4g27560 | *Arabidopsis thaliana* |
| AtUGT79B3 | At4g27570 | *Arabidopsis thaliana* |
| AtUGT79B6 | At5g54010 | *Arabidopsis thaliana* |
| AtUGT84A1 | At4g15480 | *Arabidopsis thaliana* |
| AtUGT85A1 | At1g22400 | *Arabidopsis thaliana* |
| AtUGT88A1 | At3g16520 | *Arabidopsis thaliana* |
| AtUGT89C1 | At1g06000 | *Arabidopsis thaliana* |
| AtUGT90A1 | At2g16890 | *Arabidopsis thaliana* |
| AtUGT91A1 | At2g22590 | *Arabidopsis thaliana* |
| AtUGT92A1 | At5g12890 | *Arabidopsis thaliana* |
| BoUGT79B44 | LC536578 | *Brassica oleracea* |
| BpUGAT | BAD77944 | *Bellis perennis* |
| CaUGT3 | AB443870 | *Catharanthus roseus* |
| CcUGT77B2 | MG938542 | *Crocosmia × crocosmiiflora* |
| Cm1,2RhaT | AAL06646 | *Citrus maxima* |
| Cis1,6RhaT | ABA18631 | *Citrus sinensis* |
| CsUGT78A14 | KP682360 | *Camellia sinensis* |
| CsUGT78A15 | KP682361 | *Camellia sinensis* |
| DkFGT | AB435084 | *Diospyros kaki* |
| F3GalTase | AAD55985 | *Petunia hybrida* |
| FaGT1 | AAU09442 | *Fragaria × ananassa* |
| FaGT6 | ABB92748 | *Fragaria × ananassa* |
| FaGT7 | ABB92749 | *Fragaria × ananassa* |
| FeF3G6RhaT | LC312144 | *Fagopyrum esculentum* |
| GeUGT73F1 | AB098614 | *Glycyrrhiza echinata* |
| GmF3G2Gt | LC017844 | *Glycine max* |
| GmF3G6RhaT | BAN91401 | *Glycine max* |
| GRMZM2G110511 | AY082660 | *Zea mays* |
| GRMZM2G168474 | NM_001111547 | *Zea mays* |
| Gt5GT7 | BAG32255 | *Gentiana triflora* |
| Ib3GGT | EF108571 | *Ipomoea batatas* |
| Ip3GGT | BAD95881 | *Ipomoea nil* |
| Iris5GT | BAD06874 | *Iris hollandica* |
| LeABRT2 | LC131336 | *Lobelia erinus* |
| LeABRT4 | LC131337 | *Lobelia erinus* |
| MaFGRT | KT324624 | *Morus alba* |
| MdUGT71B1 | MDP0000806350 | *Malus × domestica* |
| MdUGT75B1 | MDP0000545122 | *Malus × domestica* |
| Perilla5GT | AB013596 | *Perilla frutescens* |
| Petunia 3RT | CAA50376 | *Petunia hybrida* |
| PfF7GAT | AB362992 | *Perilla frutescens* |
| Ph3RT | X71059 | *Petunia hybrida* |
| Ph5GT | AB027455 | *Petunia hybrida* |
| PhUGT79B31 | LC387490 | *Petunia hybrida* |
| PpUGT78B | ppa005162m | *Prunus persica* |
| SbF7GlcT | SbF7GlcT | *Scutellaria baicalensis* |
| SlF7GAT | BAG31946 | *Scutellaria laeteviolacea* |
| Torenia5GT | BAC54093 | *Torenia hybrid* |
| UBGT | AB031274 | *Scutellaria baicalensis* |
| Va5GT | KF996717 | *Vitis amurensis* |
| Verbena 5GT | AB013598 | *Verbena hybrida* |
| VvGT1 | AB047092 | *Vitis vinifera* |
| VvGT5 | AB499074 | *Vitis vinifera* |
| VvGT6 | AB499075 | *Vitis vinifera* |

**Table S2.** Primers used for RT-qPCR analysis.

| **Gene** | **Primer** | **Sequence** |
| --- | --- | --- |
| qPCR-MrUFGT | Forward | CACATGATGCTGTTGGAGTGTTT |
|  | Reverse | CTCCCTTCTTTGTGAAGATCCCA |
| qPCR-MrUGT72B67 | Forward | AGTTCAACGACAACGGCCTA |
|  | Reverse | GCCAACTCAGAGAGTGCCTT |

**Table S3.** Primers used for gene clone and expression vector construction. Sequences of restriction sites are underlined.

| **Gene** | **Primer** | **Sequence** |
| --- | --- | --- |
| MrUFGT | Forward | ATGCCTTCTTCCACTACTGACTC |
|  | Reverse | TTATTTGTTGTCCTTTGCTATATCTAT |
| MrUGT72B67 | Forward | ATGGAATCAACACACACACAAGCATC |
|  | Reverse | TTACTTTTTGTTCTTCCACTTGAGAGC |
| MrUFGT-pET | Forward | gccatggctgatatcGGATCCATGCCTTCTTCCACTACTGACTC |
|  | Reverse | gtggtggtggtggtgCTCGAGTTTGTTGTCCTTTGCTATATCTAT |
| MrUGT72B67-pET | Forward | gccatggctgatatcGGATCCATGGAATCAACACACACACAAG |
|  | Reverse | gtggtggtggtggtgCTCGAGCTTTTTGTTCTTCCACTTGAGA |

**Table S4.** Primers for site-directed mutagenesis.

| **Gene** | **Sequence** |
| --- | --- |
| MrUFGT Q375H R1 | GTGGTCGCCAAAGAATGGCCTGCAAATCATAGGCACCCCG |
| MrUFGT Q375H F1 | GGCCATTCTTTGGCGACCACAGGTTGAACGGGCGGATGGT |
| MrUGT72B67 Q391H R1 | AACATAACCGCATTCATTTTGTGCTCGGCGTATAGCGGCC |
| MrUGT72B67 Q391H F1 | GGCCGCTATACGCCGAGCACAAAATGAATGCGGTTATGTT |

**Table S5.** Primers for pGreenII0029 62-SK vector constructions. Sequences of restriction sites are underlined.

| **Gene** | **Primer** | **Sequence** |
| --- | --- | --- |
| SK-MrUFGT | Forward | cgctctagaactagtGGATCCATGCCTTCTTCCACTACTGACTC |
|  | Reverse | gataagcttgatatcGAATTCTTATTTGTTGTCCTTTGCTATATCTAT |
| SK-MrUGT72B67 | Forward | cgctctagaactagtGGATCCATGGAATCAACACACACACAAGCATC |
|  | Reverse | gataagcttgatatcGAATTCTTACTTTTTGTTCTTCCACTTGAGAGC |

**Table S6.** Information of UGTs distributed on unmapped scaffold.

| **Gene** | **Group** | **Distribution** |
| --- | --- | --- |
| Mr0G015471 | E | RXIC02000424.1 |
| Mr0G007702 | E | RXIC02000188.1 |
| Mr0G007703 | E | RXIC02000188.1 |
| Mr0G007704 | E | RXIC02000188.1 |
| Mr0G007705 | E | RXIC02000188.1 |
| Mr0G022764 | E | RXIC02000460.1 |
| Mr0G022765 | E | RXIC02000460.1 |
| Mr0G022766 | E | RXIC02000460.1 |
| Mr0G007697 | H | RXIC02000187.1 |
| Mr0G007698 | H | RXIC02000187.1 |
| Mr0G007268 | G | RXIC02000164.1 |
| Mr0G025561 | G | RXIC02000485.1 |
| Mr0G025562 | G | RXIC02000485.1 |

**Table S7.** Gene duplication events in *MrUGT* gene family. WGD, whole-genome duplication; TD, tandem duplication; DSD, dispersed duplication; PD, proximal duplication.

| **No.** | **Duplicate 1** | **Duplicate 2** | **Group** | **Type** | **E-value** | **No.** | **Duplicate 1** | **Duplicate 2** | **Group** | **Type** | **E-value** |
| --- | --- | --- | --- | --- | --- | --- | --- | --- | --- | --- | --- |
| 1 | Mr1G005704 | Mr5G023765 | M | WGD | 0 | 1 | Mr8G028159 | Mr1G022059 | C | DSD | 1.9E-115 |
| 2 | Mr2G000905 | Mr3G014797 | H | WGD | 3.1E-160 | 2 | Mr2G018501 | Mr8G003331 | A | DSD | 5.65E-87 |
| 3 | Mr3G015805 | Mr5G017601 | E | WGD | 1.9E-127 | 3 | Mr4G010558 | Mr3G012011 | H | DSD | 4.52E-90 |
| 4 | Mr1G019344 | Mr1G019345 | H | TD | 0 | 4 | Mr8G002336 | Mr8G003331 | A | DSD | 2.49E-33 |
| 5 | Mr7G022693 | Mr7G022692 | L | TD | 0 | 5 | Mr8G012797 | Mr1G022059 | C | DSD | 2.45E-93 |
| 6 | Mr5G011880 | Mr5G011881 | B | TD | 1.6E-135 | 6 | Mr3G011171 | Mr3G009799 | G | DSD | 0 |
| 7 | Mr1G027745 | Mr1G027744 | L | TD | 0 | 7 | Mr8G019535 | Mr7G024840 | L | DSD | 0 |
| 8 | Mr8G010072 | Mr8G010073 | J | TD | 0 | 8 | Mr7G013561 | Mr7G023667 | F | DSD | 7.69E-26 |
| 9 | Mr3G014796 | Mr3G014795 | H | TD | 0 | 9 | Mr5G003419 | Mr5G003415 | L | PD | 0 |
| 10 | Mr2G011696 | Mr2G011695 | K | TD | 3.1E-125 | 10 | Mr7G008228 | Mr7G008223 | G | PD | 0 |
| 11 | Mr8G010075 | Mr8G010076 | J | TD | 9E-106 | 11 | Mr2G016166 | Mr2G016168 | O | PD | 1.73E-85 |
| 12 | Mr2G025727 | Mr2G025728 | D | TD | 0 | 12 | Mr2G022300 | Mr2G022302 | G | PD | 0 |
| 13 | Mr5G025050 | Mr5G025051 | C | TD | 2.2E-144 | 13 | Mr1G025744 | Mr1G025742 | E | PD | 1.14E-87 |
| 14 | Mr2G011693 | Mr2G011692 | K | TD | 0 | 14 | Mr5G011888 | Mr5G011891 | B | PD | 2.3E-93 |
| 15 | Mr3G014795 | Mr3G014794 | H | TD | 0 | 15 | Mr2G011713 | Mr2G011710 | K | PD | 6.7E-132 |
| 16 | Mr2G025729 | Mr2G025730 | D | TD | 4.98E-79 | 16 | Mr5G003481 | Mr5G003477 | L | PD | 0 |
| 17 | Mr1G017649 | Mr1G017648 | E | TD | 0 | 17 | Mr5G003415 | Mr5G003410 | L | PD | 3.31E-27 |
| 18 | Mr8G027413 | Mr8G027412 | E | TD | 1.2E-144 | 18 | Mr5G024791 | Mr5G024793 | G | PD | 1.3E-158 |
| 19 | Mr2G025728 | Mr2G025729 | D | TD | 1.16E-77 | 19 | Mr5G011889 | Mr5G011891 | B | PD | 2.06E-74 |
| 20 | Mr7G015242 | Mr7G015241 | E | TD | 3.1E-180 | 20 | Mr5G003531 | Mr5G003525 | M | PD | 0 |
| 21 | Mr8G027414 | Mr8G027413 | E | TD | 0 | 21 | Mr5G003480 | Mr5G003476 | L | PD | 0 |
| 22 | Mr2G025731 | Mr2G025732 | D | TD | 0 | 22 | Mr8G002343 | Mr8G002346 | A | PD | 0 |
| 23 | Mr4G022362 | Mr4G022363 | E | TD | 0 | 23 | Mr7G022005 | Mr7G022003 | E | PD | 2.76E-41 |
| 24 | Mr6G024468 | Mr6G024467 | G | TD | 0 | 24 | Mr7G022698 | Mr7G022693 | L | PD | 8.4E-122 |
| 25 | Mr1G014008 | Mr1G014007 | B | TD | 1.1E-157 | 25 | Mr6G024794 | Mr6G024796 | G | PD | 9.43E-55 |
| 26 | Mr7G028521 | Mr7G028522 | D | TD | 0 | 26 | Mr6G024469 | Mr6G024467 | G | PD | 7E-111 |
| 27 | Mr2G025730 | Mr2G025731 | D | TD | 0 | 27 | Mr3G015803 | Mr3G015806 | G | PD | 8.32E-14 |
| 28 | Mr8G002346 | Mr8G002347 | A | TD | 0 | 28 | Mr5G003421 | Mr5G003419 | L | PD | 0 |
| 29 | Mr3G026042 | Mr3G026043 | L | TD | 2.92E-52 | 29 | Mr3G015804 | Mr3G015807 | G | PD | 1.34E-92 |
| 30 | Mr7G015243 | Mr7G015242 | E | TD | 0 | 30 | Mr2G011714 | Mr2G011711 | K | PD | 0 |
| 31 | Mr4G020672 | Mr4G020673 | O | TD | 0 | 31 | Mr2G009175 | Mr2G009173 | L | PD | 6.4E-143 |
| 32 | Mr8G002347 | Mr8G002348 | A | TD | 0 | 32 | Mr2G009178 | Mr2G009173 | L | PD | 2.38E-32 |
|  |  |  |  |  |  | 33 | Mr2G009177 | Mr2G009175 | L | PD | 3.2E-101 |
|  |  |  |  |  |  | 34 | Mr5G011877 | Mr5G011880 | B | PD | 1.1E-164 |
|  |  |  |  |  |  | 35 | Mr2G009173 | Mr2G009170 | L | PD | 0 |
|  |  |  |  |  |  | 36 | MrUGT78R1 | MrUGT78R2 | F | PD | 0 |

**Table S8.** *MrUGT* genes significantly induced by UV-B treatment (log_2_FC > 1, *p* < 0.05).

| **Day** | **Group** | **GeneID** | **log_2_FC** | ***p* value** |
| --- | --- | --- | --- | --- |
| 2d | A | Mr8G002346 | 1.090 | 0.005 |
|  | B | Mr5G011877 | 1.110 | 0.001 |
|  | E | Mr1G017651 | 2.939 | 0.013 |
|  | F | MrUFGT | 3.095 | 0.003 |
|  | F | MrUGT78R2 | 6.475 | 0.001 |
|  | G | Mr5G024791 | 2.102 | 0.001 |
|  | L | Mr3G026042 | 1.396 | 0.000 |
| 6d | A | Mr8G002346 | 2.017 | 0.015 |
|  | D | Mr2G025728 | 1.494 | 0.026 |
|  | F | MrUGT78R1 | 2.410 | 0.005 |
|  | F | MrUGT78W1 | 1.087 | 0.010 |
|  | F | MrUFGT | 3.458 | 0.002 |
|  | F | MrUGT78R2 | 8.405 | 0.023 |
|  | G | Mr6G024467 | 1.172 | 0.001 |
|  | G | Mr6G024796 | 1.158 | 0.009 |
|  | G | Mr5G024793 | 2.253 | 0.018 |
|  | L | Mr3G026042 | 1.652 | 0.000 |
